# Typical carcinoid in right middle lobe of pulmonary hypoplasia

**DOI:** 10.1186/s40792-023-01718-4

**Published:** 2023-07-31

**Authors:** Yasuaki Kubouchi, Shunsuke Kojima, Wakako Fujiwara, Tatsuya Miyamoto, Shinji Matsui, Takashi Ohno, Tomohiro Haruki, Hiroshige Nakamura

**Affiliations:** Department of Surgery Division of General Thoracic Surgery and Breast and Endocrine Surgery Faculty of Medicine, Tottori University, 86 Nishicho, Yonago, Tottori 683-8503 Japan

**Keywords:** Pulmonary hypoplasia, Typical carcinoid, Lung cancer

## Abstract

**Background:**

Pulmonary typical carcinoid occurring in hypoplasia of the right middle lobe is very rare.

**Case presentation:**

A routine examination's chest X-ray revealed an abnormal shadow in the right middle lung field of an 82-year-old Japanese woman. A chest computed tomography scan showed a solid 2.5 × 2.0-cm nodule in the very small right middle lobe. A trans-bronchial lung biopsy of the mass in the right middle lobe was performed; it revealed atypical cells with round nuclei growing in multiple foci, and immunostaining was positive for chromogranin A, synaptophysin and CD56, suggesting pulmonary carcinoid. The preoperative clinical diagnosis of primary lung cancer, cT1cN0M0 stage IA3 was considered. A right middle lobectomy and mediastinal lymph node dissection were performed by video-assisted thoracic surgery. Intraoperatively, the middle lobe of the right lung was very small, with 1- to 2-mm-dia. pulmonary arteries and veins that were considered hypoplastic. The final histopathological diagnosis was typical carcinoid, pT2aN0M0 stage IB based on the presence of pleural invasion.

**Conclusions:**

Including the present patient, only nine cases of lung cancer occurring within pulmonary hypoplasia have been reported, most of which were typical carcinoid.

## Background

The frequency of pulmonary hypoplasia has been reported to be < 0.01% [1, 2], and reports of lung cancer combined with pulmonary hypoplasia are very rare. We describe a rare case of typical carcinoid occurring in right middle lobe hypoplasia.

## Case presentation

**Fig. 1:**
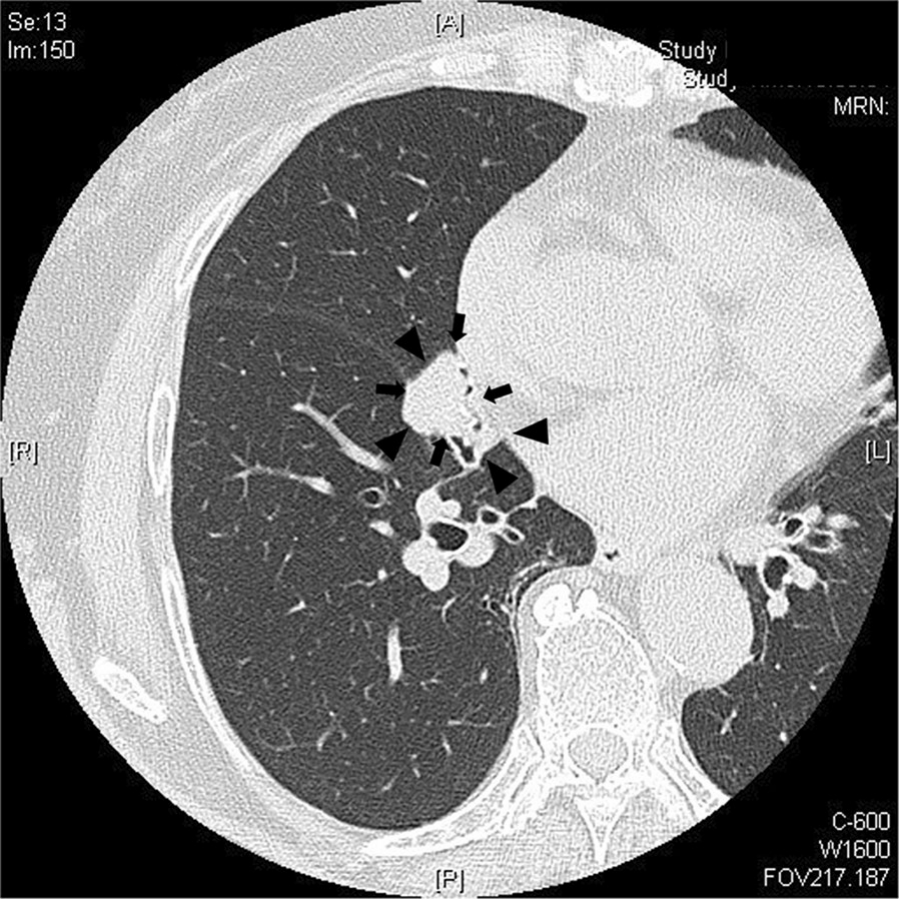
Chest CT showed a solid 2.5 × 2.0-cm nodule in the right middle lobe. Arrows indicate tumors and arrowheads indicate hypoplastic lungs. The right middle lobe was very small and was a hypoplastic lung. Most of the right middle lobe was occupied by tumor

**Fig. 2:**
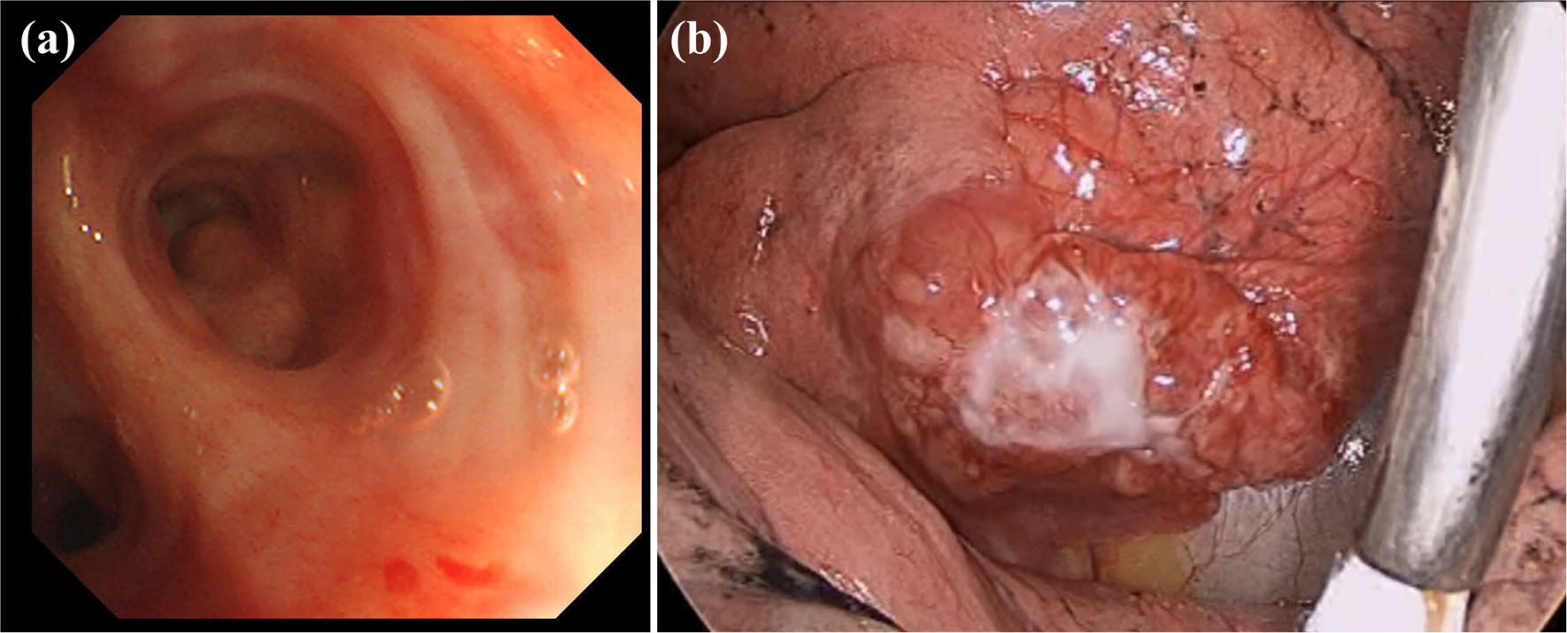
**a** Bronchoscopy showed no abnormal findings in the bifurcation of the right middle lobe bronchus. **b** Thoracoscopy revealed that the tumor occupied most of the right middle lobe

A routine examination's chest X-ray revealed an abnormal shadow in the right middle lung field of a never-smoker, 82-year-old Japanese woman. She had a medical history of atrial fibrillation and stomach ulcer. A chest computed tomography (CT) scan showed a solid 2.5 × 2.0-cm nodule in the very small right middle lobe (Fig. 1). Positron emission tomography (PET) showed an accumulation of 18F-fluorodeoxyglucose (FDG) in the nodule, with a maximum standardized uptake value (SUVmax) of 3.68 in the early phase and 5.63 in the late phase. Bronchoscopy showed patency of B4 and B5 up to subsegmental bronchi and no abnormal bifurcation (Fig. 2a).

A trans-bronchial lung biopsy (TBLB) of the mass in the right middle lobe was performed; it revealed atypical cells with round nuclei growing in multiple foci, and immunostaining was positive for chromogranin A, synaptophysin and CD56, suggesting pulmonary carcinoid. The preoperative clinical diagnosis of primary lung cancer, cT1cN0M0 stage IA3 was considered.

**Fig. 3:**
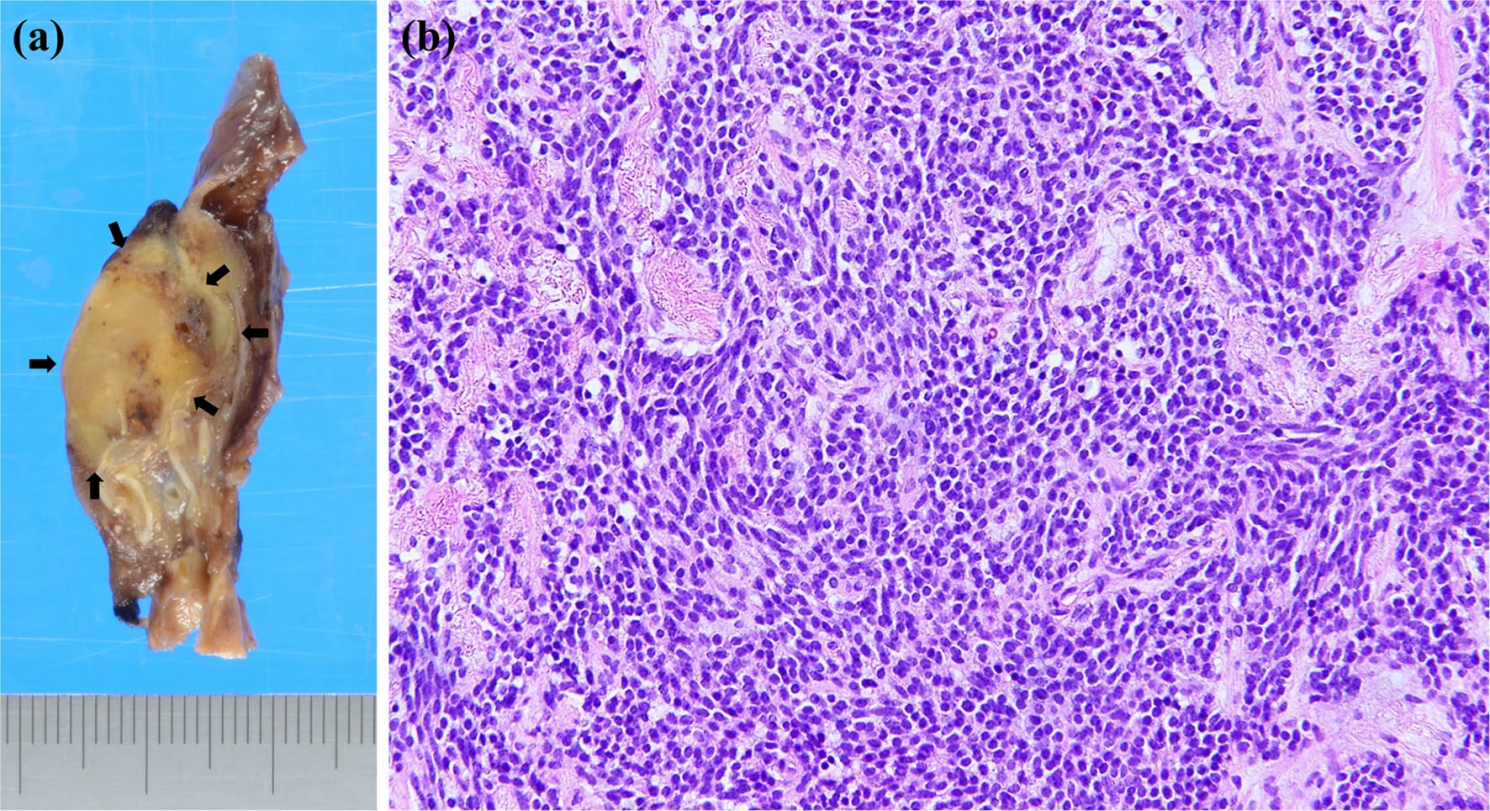
**a** Macroscopic findings of a formalin-inflated resection specimen. Macroscopic findings showed a 2.8 × 1.3 × 1.1-cm-dia. tumor in the right middle lobe. Tumor extent is indicated by arrows. **b** The histopathological examination revealed that the tumor was composed of a proliferation of atypical cells with round or oval nuclei and stippled chromatin without mitosis or necrosis

A right middle lobectomy and mediastinal lymph node dissection were performed by video-assisted thoracic surgery (Fig. 2b). Intraoperatively, the middle lobe of the right lung was very small, and both the pulmonary arteries and veins were 1–2 mm in diameter, which was considered pulmonary hypoplasia. The size of the resected right middle lobe was 5.3 × 3.0 × 2.8 cm, and the tumor was 2.8 × 1.3 × 1.1 cm (Fig. 3a).

**Fig. 4:**
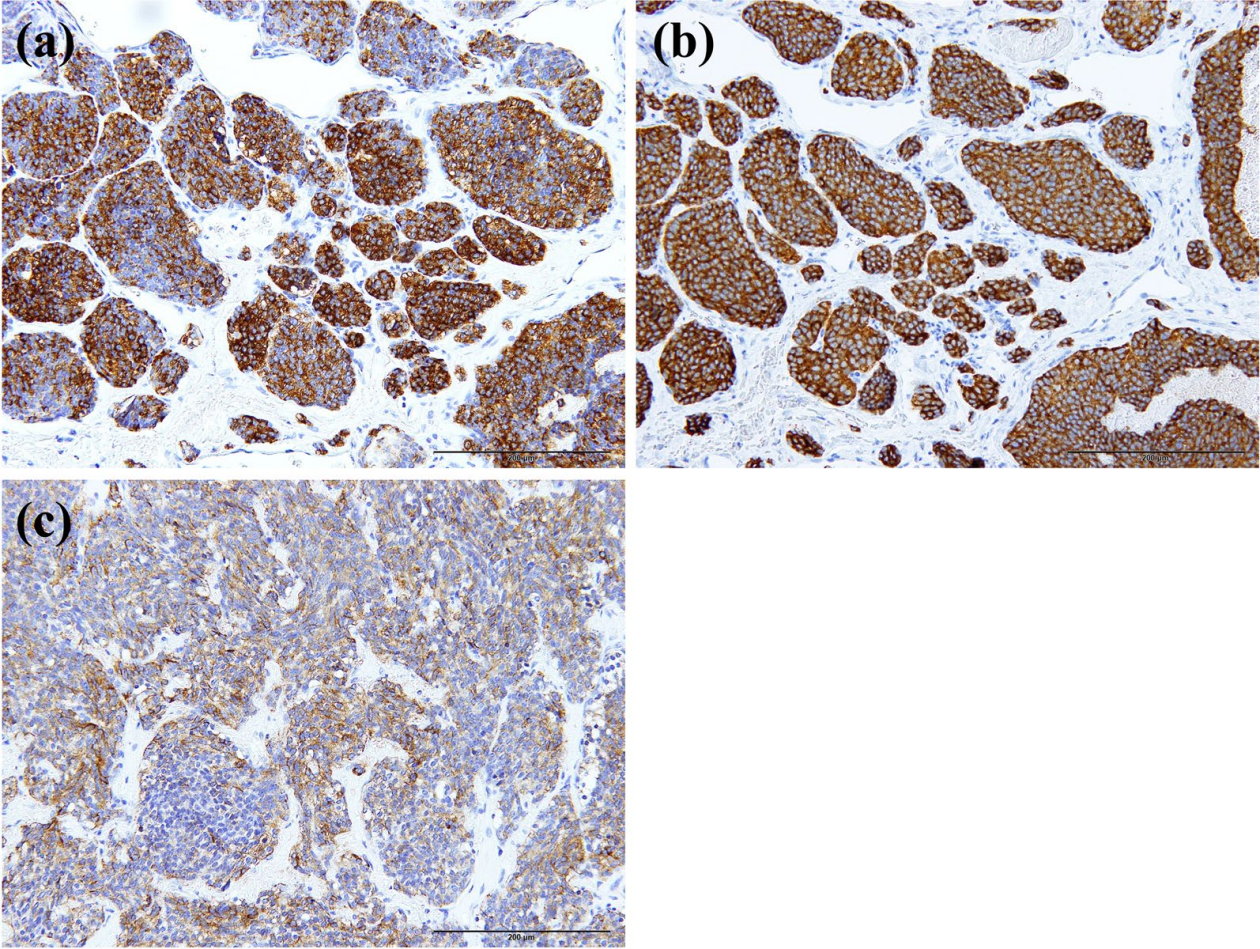
The immunohistochemical analysis demonstrated positivity for chromogranin A (**a**), synaptophysin (**b**), and CD56 (**c**)

The histopathological examination showed that the tumor was composed of a proliferation of atypical cells with round or oval nuclei and stippled chromatin. Pleural invasion of tumor cells was observed. There was no mitosis or necrosis (Fig. 3b). The immunohistochemical analysis revealed positivity for chromogranin A, synaptophysin, and CD56 (Fig. 4). The normal area of the resected lung did not show the inflammatory cell infiltration usually seen in atelectatic lung, so we consider it to be lung hypoplasia rather than atrophic lung caused by atelectasis. The final histopathological diagnosis was typical carcinoid, pT2aN0M0 stage IB. Because of the patient's advanced age, she was followed up without adjuvant chemotherapy and has survived for 5 years without recurrence.

## Discussion

Regarding the frequency of pulmonary dysplasia including hypoplasia, Krivchenya et al. [1] reported pulmonary dysplasia including hypoplasia in 0.01–0.067% of their series of autopsy cases, and Conway et al. [2] reported the rate 0.001% of delivery cases. This disorder rarely remains asymptomatic into adulthood, due to the presence of severe respiratory complications and/or congenital heart disease and gastrointestinal, genitourinary, and skeletal abnormalities in 50–85% of cases of pulmonary dysplasia including hypoplasia [3, 4]. Pulmonary dysplasia can be classified into three categories: aplasia, in which lung parenchyma, bronchi, and pulmonary vessels are absent; dysplasia, in which traces of bronchi are present but lung tissue and pulmonary vessels are absent; and hypoplasia, in which lung parenchyma, bronchi, and vessels are present but the number of alveoli is reduced and the airways and vessels are immature [1]. In the present case, there was no luminal narrowing or obstruction in the right middle lobe bronchus, and the presence of immature lung parenchyma and vessels suggested hypoplasia.

The cases of nine patients with lung cancer within pulmonary hypoplasia [5–9] including our present patient have been reported (Table 1). Interestingly, all cases were neuroendocrine tumors of right middle lobe origin, and eight of the nine cases were typical carcinoid tumors. Moreover, all of these cases were reported from Japan. Although there are reports of unknown prognosis and short observation periods, none of them have reported recurrence, and the prognosis appears to be good. Yukawa et al. speculated that neuroendocrine cells are activated to compensate for tissue remodeling in the hypoplastic lung, resulting in neuroendocrine hyperplasia, which may lead to carcinoid-precursor lesion development [9]. Motono et al. noted that if lung hypoplasia causes reactive neuroendocrine hyperplasia, then neuroendocrine tumors may occur with lung hypoplasia [8]. Although there are few reports of lung cancers developing in pulmonary hypoplasia, most of the cases are typical carcinoid, suggesting that there is some association between lung hypoplasia and carcinoid. To elucidate this, it may be necessary to pathologically clarify the state of neuroendocrine cells in the bronchial epithelium of the pulmonary hypoplasia.

**Table 1:** Summary of lung cancer developing in pulmonary hypoplasia in the right middle lobe

Case no.	First author, year	Age, years	Sex	Smoking index	Tumor size, mm	Right middle lobe max. dia., mm	Histology	*p*-Stage	Follow-up
1	Sato 2001	76	F	200	8	Unknown	Typical carcinoid	IA1	10 m, no recurrence
2	Maeshiro 2016	68	F	200	12	40	Typical carcinoid	IA2	3 m, no recurrence
3	Yoshida 2018	61	M	1440	5	50	Typical carcinoid	IA1	30 m, no recurrence
4	Yoshida 2018	60	F	0	10	Unknown	Small-cell lung cancer	IIB	84 m, no recurrence
5	Yoshida 2018	64	M	880	5	Unknown	Typical carcinoid	IA1	48 m, no recurrence
6	Yoshida 2018	68	M	900	14	Unknown	Typical carcinoid	IA2	20 m, no recurrence
7	Motono 2020	70	M	400	30	30	Typical carcinoid	IA3	Unknown
8	Yukawa 2022	70	M	960	5	60	Typical carcinoid	IA1	Unknown
9	Present case, 2023	82	F	0	28	53	Typical carcinoid	IB	60 m, no recurrence

*F* female, *M* male, *m* months

There are several surgical problems in cases of lung cancer with pulmonary hypoplasia. Because pulmonary hypoplasia is occasionally associated with abnormal bronchial and vascular structures, it is necessary to observe the bronchial lumen by bronchoscopy and to fully confirm the pulmonary arteriovenous branches by CT scanning before surgery. In addition, as in the present case, the pulmonary arteries and veins are very thin in hypoplastic lungs, and careful manipulation is necessary to avoid extraction injury. The lung parenchyma is very small in hypoplastic lungs, making it difficult to avoid touching the tumor. Lung cancer occurring in pulmonary hypoplasia is a concern because the volume of lung parenchyma is small relative to the size of the tumor, making it more likely to invasive surround tissues. However, no invasion into surrounding tissues has been reported to date, which may be related to the fact that the tumor is a typical carcinoid with good prognosis and a central bronchogenic origin.

## Conclusions

We have described a case of typical carcinoid developing in right middle lobe hypoplasia. Since previous reports suggest an associated between pulmonary hypoplasia and typical carcinoid, it will be necessary to accumulate more cases to elucidate the association.

## Data Availability

The authors declare that all the data in this article are available within the article.

## Abbreviations

CT: Computed tomography
PET: Positron emission tomography
FDG: Fluorodeoxyglucose
SUV: Standardized uptake value
TBLB: Trans-bronchial lung biopsy
